# Direct Visualization by Cryo-EM of the Mycobacterial Capsular Layer: A Labile Structure Containing ESX-1-Secreted Proteins

**DOI:** 10.1371/journal.ppat.1000794

**Published:** 2010-03-05

**Authors:** Musa Sani, Edith N. G. Houben, Jeroen Geurtsen, Jason Pierson, Karin de Punder, Maaike van Zon, Brigitte Wever, Sander R. Piersma, Connie R. Jiménez, Mamadou Daffé, Ben J. Appelmelk, Wilbert Bitter, Nicole van der Wel, Peter J. Peters

**Affiliations:** 1 Division of Cell Biology-B6, Netherlands Cancer Institute–Antoni van Leeuwenhoek Hospital (NKI-AVL), Amsterdam, the Netherlands; 2 Department of Medical Microbiology and Infection Control, VU University Medical Centre, Amsterdam, the Netherlands; 3 Department of Medical Oncology, VU University Medical Centre, Amsterdam, the Netherlands; 4 CNRS, Institut de Pharmacologie et de Biologie Structurale, Département Mécanismes Moléculaires des Infections Mycobactériennes, Toulouse, France; 5 Kavli Institute of Nanoscience, Delft University of Technology, Delft, the Netherlands; University of Washington, United States of America

## Abstract

The cell envelope of mycobacteria, a group of Gram positive bacteria, is composed of a plasma membrane and a Gram-negative-like outer membrane containing mycolic acids. In addition, the surface of the mycobacteria is coated with an ill-characterized layer of extractable, non-covalently linked glycans, lipids and proteins, collectively known as the capsule, whose occurrence is a matter of debate. By using plunge freezing cryo-electron microscopy technique, we were able to show that pathogenic mycobacteria produce a thick capsule, only present when the cells were grown under unperturbed conditions and easily removed by mild detergents. This detergent-labile capsule layer contains arabinomannan, α-glucan and oligomannosyl-capped glycolipids. Further immunogenic and proteomic analyses revealed that *Mycobacterium marinum* capsule contains high amounts of proteins that are secreted via the ESX-1 pathway. Finally, cell infection experiments demonstrated the importance of the capsule for binding to cells and dampening of pro-inflammatory cytokine response. Together, these results show a direct visualization of the mycobacterial capsular layer as a labile structure that contains ESX-1-secreted proteins.

## Author Summary

The genus *Mycobacterium* contains a number of important pathogens, such as *Mycobacterium tuberculosis*. The highly characteristic cell envelope of these bacteria plays a crucial role in the infection process. The most apparent difference with other bacteria is the recently described outer membrane composed of unique (glyco)lipids. However, on top of this membrane mycobacteria also have an ill-defined capsular layer. In this paper, we studied this capsular layer using different electron microscopy techniques and mass spectrometry. Using close to native state preparation method, we show that both pathogenic and non-pathogenic mycobacteria have a labile capsular layer that covers the outer membrane. This capsular layer, in addition to containing arabinogalactan, glycan and mannose-containing glyco-lipids, also surprisingly contains a large amount of ESX-1-secreted proteins in *Mycobacterium marinum*. Furthermore, we also show that the capsule plays a role in the binding of macrophages and the induction of cytokines. Collectively, these results show for the first time that the capsule can be visualized on both pathogenic and non-pathogenic mycobacteria. In addition, growing mycobacteria under standard laboratory conditions in the presence of detergent with agitation promotes capsular shedding and influences the biological characteristics of the bacteria.

## Introduction

Mycobacteria are the causative agent of tuberculosis and other chronic diseases such as leprosy and causes about 1.7 million deaths annually. The interaction of *Mycobacterium tuberculosis* with its macrophage host cell is largely dictated by its unique cell envelope components that are able to elicit immuno-modulatory responses similar to that of the pathogen [1].

The mycobacterial cell envelope has a complex structure composed of a typical phospholipid bilayer plasma membrane (PM), an outer membrane and an outermost layer known as the capsule in the case of pathogenic species [2]. The *myco*bacterial outer *membrane* henceforth referred to as ‘mycomembrane’ is mainly composed of long chain (C60-C90) mycolic fatty acids with free intercalating glycolipids and is covalently linked to the arabinogalactan-peptidoglycan layer [3],[4]. The chemical nature of mycomembrane has been examined in detail [4] and recent cryo-transmission electron microscopy (EM) data shows that it organizes into a structure analogous to the outer membrane of Gram-negative bacteria [5],[6]. The structural organization of this highly insoluble matrix is invariable among different mycobacterial species and is responsible for the low permeability of the mycobacterial cell envelope [4].

The capsule is visible as an electron transparent zone (ETZ) surrounding the mycobacterial cell envelope [2],[7] in conventional EM preparations [8],[9]. This layer was not, however, observed by cryo-EM [5],[6], questioning somehow its existence. This layer, mainly composed of polysaccharides, proteins and small amounts of lipids, is considered to have a different molecular composition in pathogenic and non-pathogenic species [2],[10],[11]. The presence of the capsular layer is influenced by the culturing conditions used; laboratory cultures commonly grown in the presence of detergent with agitation to prevent clumping [12] usually shed the capsule into the medium [13],[14].

In the present study, we adopt a close to native-state approach to study the mycobacterial cell envelope and demonstrate that mycobacteria express a distinctive outer layer. We have characterized the mycobacterial capsule layer by immunological and proteomic analyses and showed that some mycobacteria have ESX-1 secreted proteins in their capsule layer. ESX-1 encodes a type VII secretion system that mediates the secretion of potent T cell antigens such as ESAT-6 (EsxA) and CFP-10. Furthermore, the presence of a capsule not only enhanced the *Mycobacterium*-macrophage interaction but also dampened the pro-inflammatory cytokine response.

## Results/Discussion

### Cell envelope organization of mycobacterial cells

The ultra-structure of the mycobacterial cell envelope was investigated using 30 nm vitreous sections, as previously described [15]. As a control for structural preservation of the envelope morphology, we also examine vitreous sections of the Gram-negative bacterium *Shigella flexneri* and the Gram-positive bacterium *Staphylococcus epidermidis*. The width of the PM and outer membrane or cell wall in both species are consistent with previous results [16],[17] (Figure S1, Table S1). The vitreous sections of four mycobacterial species [*M. tuberculosis*, *M. marinum*, *Mycobacterium bovis* Bacille Calmette-Guérin (BCG) and *Mycobacterium smegmatis*] examined appeared similar and show a well preserved cell envelope composed of a PM and a mycomembrane with a bilayer profile (Figure S1C, S2 and S3). Although the intensity of the mycobacterial PM is similar to that of *S. flexneri* and *S. epidermidis*, the width (≈7 nm) is at least 16% wider (Table S1). The mycomembrane, with an apparent thickness of ≈8.3 nm is also thicker than the 6.8 nm thick outer membrane of *S. flexneri*. This slight increase in thickness is similar to recently published data [5],[6].

Cryo-electron tomography analysis was performed to determine the 3D architecture of the envelope (Figure S1D-F). In the reconstructed slices of *M. smegmatis* cell envelope, two additional layers are present in the periplasmic space. These layers are similar to those observed as L1 and L2 in [5] and as the granular layer and the medial wall zone referred to in [6]. The L1 layer appears immediately after the plasma membrane while the L2 layer appears closely apposed to the mycomembrane and may possibly be the peptidoglycan/arabinogalactan matrix. Taken together, these findings show that the cell envelope morphology of the mycobacteria is structurally related to, but more complex than that of the Gram-negative bacteria.

### Visualization of the mycobacterial capsular layer and its disruption by Tween and agitation

The capsule, being a substantial component of the cell envelope, should be visible in vitreous sections as an extra layer extending from the mycomembrane. Yet, in our analyses and previous studies [5],[6], such a layer is not visible (Figure S1C, S3). We reasoned that the detection of this layer may have been obscured by the presence of the dextran cryoprotectant, which may have an electron density similar to that of the capsule. Alternatively, the capsule might have been removed by growing the cells in the presence of detergent and agitation in order to prevent clumping [2],[13],[14].

**Figure 1:**
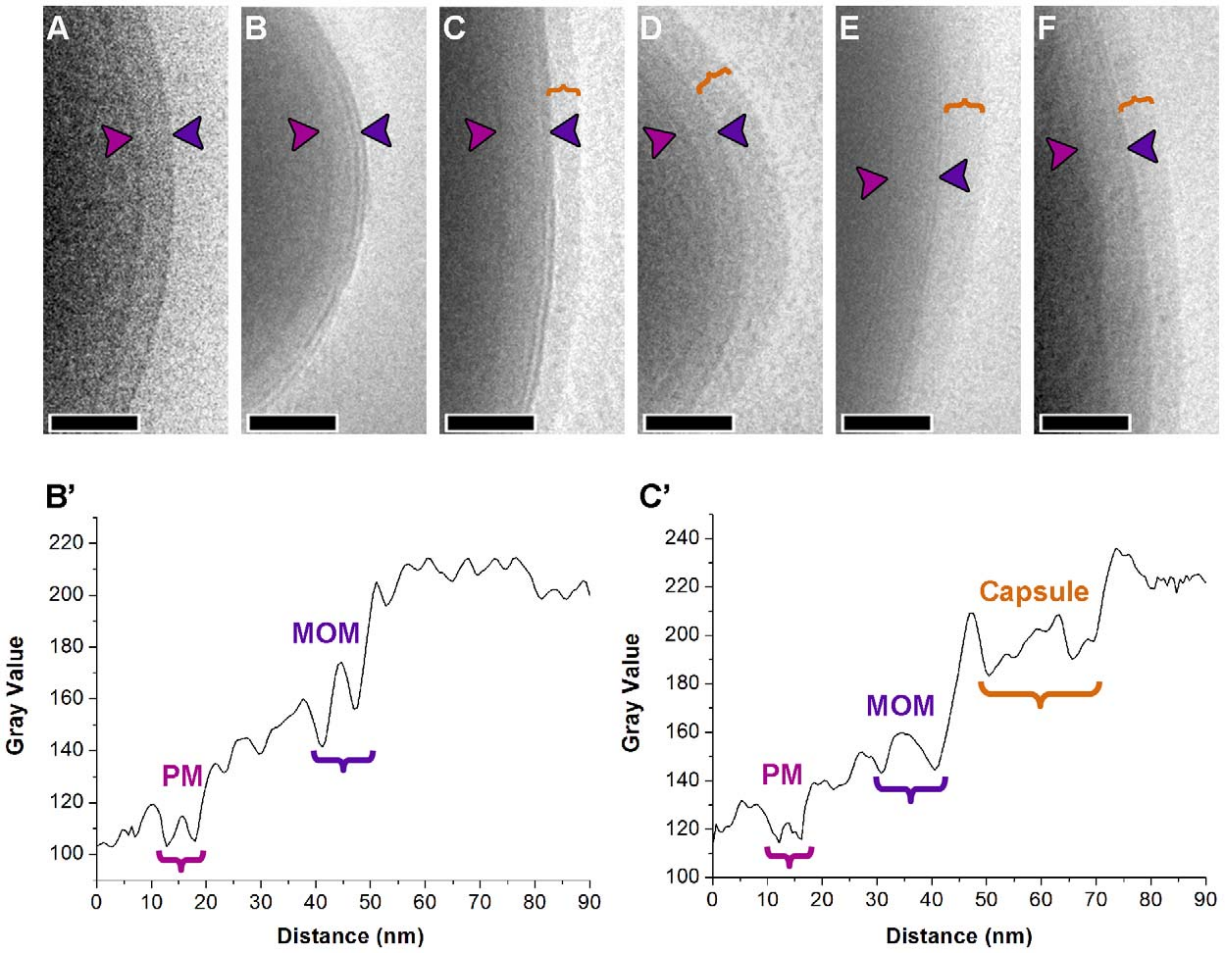
Visualization of the capsule in its native state. Cryo electron micrographs of intact *S. flexneri* cell plunge frozen (A) depicts the typical cell envelope profile using this method of sample preparation. (B) *M. smegmatis* cells grown with both chemical and mechanical perturbation shows a cell envelope with morphology similar to *S. flexneri*. Similar cells cultured in an unperturbed state before freezing (C) shows the presence of an extra layer (bracket) surrounding the mycomembrane. Corresponding layers are also observed surrounding the mycomembranes of *M. tuberculosis* (D), *M. marinum* (E), and *M. bovis* BCG (F). The density profiles in B' and C' were obtained from images corresponding to B and C respectively. Images were averaged over a width of 75 pixels. The outermost layer varies in thickness from negligible to considerable (40 nm; see Table S1) indicating the unstable nature of this layer. Arrow heads point to plasma membrane (PM; magenta) and outer membrane/mycomembrane (MOM; blue). Scale bars: 100 nm.

To investigate this, mycobacterial cells were cultured with or without Tween-80 and agitation and subsequently frozen in a close to native-state by the plunge freezing method [18] for direct visualization by EM (Figure 1). This method does not rely on the use of cryoprotectants and allows intact cells to be frozen in their medium of culture. The Gram-negative bacterium *S. flexneri* was used as a control (Figure 1A). When grown without any perturbation, all mycobacterial species examined, showed a thick outermost capsule-like layer (Figure 1C to 1F). To our knowledge, this is the first time this layer is visualized in a close to native state surrounding both pathogenic and nonpathogenic mycobacterial species. In comparison, cells grown with perturbation show this layer to be partially or completely removed (Figure 1B), meaning that growing mycobacteria under routine culturing conditions involving perturbation promotes shedding of this layer.

**Figure 2:**
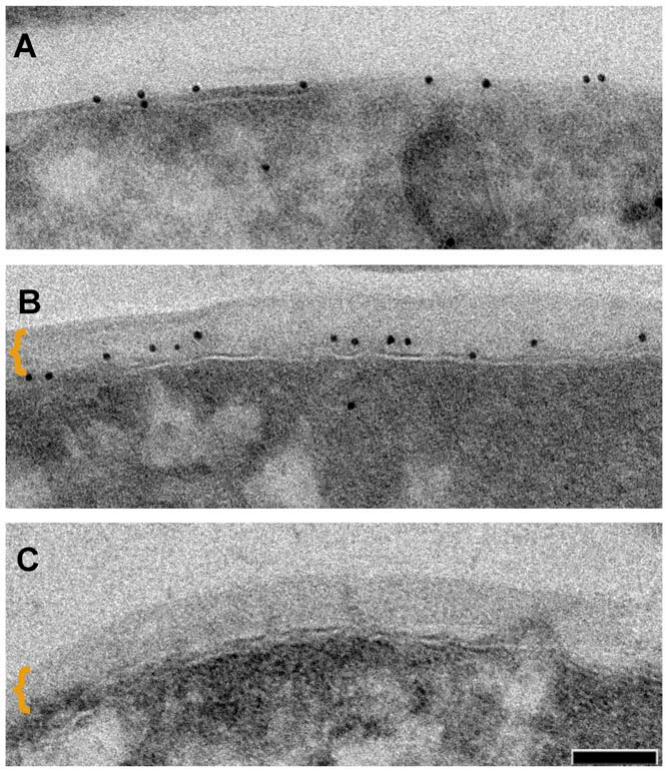
Distinction between the mycomembrane and the outermost layer. Immuno-labeling of outer membrane porin OmpATb on thin sections of *M. smegmatis* overexpressing OmpATb (mechanically agitated and detergent treated culture) (A) and non-perturbed culture (B) as compared to *M. smegmatis* wt (C). Outermost layer indicated by bracket. Scale bar: 50 nm.

To demonstrate that this outermost layer is distinct from the mycomembrane, we sought to localize OmpATb porin known to be present in the mycomembrane [19] by labeling fixed cryo-sections of *M. smegmatis* cells over-expressing this protein [20] with anti-OmpATb serum and probed with protein-A attached to 10 nm gold. When grown in the presence of perturbation, the mycomembrane was efficiently labeled with the antibody (Figure 2A). In comparison, cells grown in the absence of the detergent maintain the outermost layer, though the OmpATb antibody only specifically labeled the underlying mycomembrane (Figure 2B). Parental *M. smegmatis* strain, naturally devoid of OmpATb, cultured under unperturbed state lacks any labeling, demonstrating the specificity of this antibody (Figure 2C). These results indicate that the outermost layer removed by perturbation is distinct and not part of the mycomembrane.

### Characterization of the mycobacterial capsular layer by immuno gold-EM

**Figure 3:**
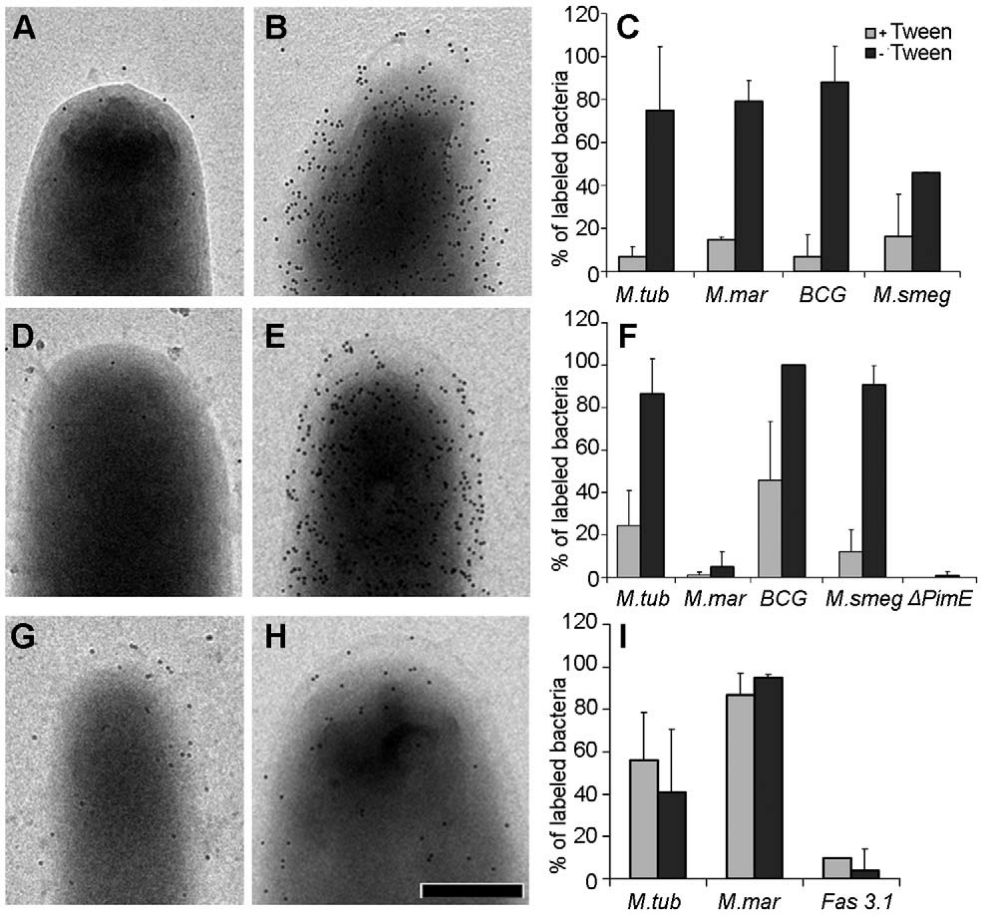
Effect of detergent on the localization of capsular components and the detection of ESX-1 proteins. *M. smegmatis* (A and B), *M. tuberculosis* (D and E) and *M. marinum* (G and H) were grown under both perturbed (A,D and G) and unperturbed conditions (B,E and H), fixed and probed with anti-α-glucan (A–B) and anti PIM/capLAM (D–E) to demonstrate that this extra layer is of capsular origin. (G–H) demonstrates the presence of anti-EspE on bacterial surface/capsule. Histogram (C,F and I) respectively displays the variations in anti glucan, anti PIM/capLAM and anti-EspE labeling of bacteria due to change in the culturing conditions using *M. smegmatis* ΔPimE k.o. and *M. marinum* fas 3.1 *eccCb1* mutant as controls. The ordinate (defined as percentage of bacteria with ≥10 gold particles) represents the average ± standard error of labeled cells from 3 independent experiments. Scale bars: 250 nm.

To confirm that the observed outermost layer is indeed the capsule, immuno gold-EM analysis of whole bacteria using antisera against known capsular components was performed. Whole bacteria were immobilized on EM grids and incubated with specific antibodies and gold probes. This method also allowed us to visualize and quantify the different components. Since α-glucan is described to be the major capsular polysaccharide [2],[11], we first investigated this using an α-glucan-specific monoclonal antibody [21]. The bacterial surface of cells grown in unperturbed state was distinctly labeled homogenously with this antibody (Figure 3), whereas cells grown in perturbed state showed weak or no labeling. Fixation of the cells was important for efficient labeling, since the same unperturbed culture without any fixation showed reduced or weak labeling. Non-acylated arabinomannan (AM) is also a described component of the capsule [11],[22]. Using antibodies directed against arabinomannan structural motifs, our experiments confirmed the presence of AM in the capsule (Figure S4) Subsequently, we tried to localize the glycolipid phosphatidylinositol mannosides (PIMs), which is possibly associated with the capsule [23]. For this we used an antibody that on *M. smegmatis* specifically recognizes the PIM_6_ epitope, as it does not interact with *M. smegmatis pimE* mutant (Figure 3F) [24],[25]. The antibody labeled the surface of intact cells very efficiently, supporting the presence of specific PIM epitopes on *M. smegmatis* and possibly other mannoconjugates and PIM on the pathogenic mycobacteria except for *M. marinum* which is poorly labeled with this antibody. Importantly, all of the antibodies significantly labeled less when the bacteria were grown agitated in the presence of detergent (Figure 3A, 3D and S4). Agitation in the absence of detergent contributes to the erosion of the capsule albeit lesser than when detergent is also included, conversely, the addition of detergent alone also shows less capsular disruption, thus the effect is additive.

In addition to glycans and glycolipids, the capsule also contains proteins [2],[10], although these are generally not well described. One putative capsular protein recently described by Carlsson *et al* [26] is the ESX-1-associated protein EspE [formerly known as Mh3864 [27]], which together with EspB is also secreted by ESX-1 encoded type VII secretion system [26],[28]. To investigate EspE localization, whole cells were labeled with anti-EspE antibody which homogenously labeled the surface of *M. marinum* and *M. tuberculosis.* A preference of EspE for the polar region as previously reported [26] was not observed (Figure 3G-I). The localization of EspE in a *M. marinum* strain (Fas 3.1) with a transposon insertion in *eccCb1* [formerly known as Mh1784 [27]] which blocks ESX-1-dependent secretion (van der Sar *et al*, in preparation), showed no specific labeling, demonstrating both the specificity of the antibody used and the ESX-1 dependence of EspE export. Finally, EspE labeling was not strongly affected by Tween-80, suggesting that this protein is more tightly associated with the cell envelope.

### Capsule of *M. marinum* contains secretion specific proteins

**Figure 4:**
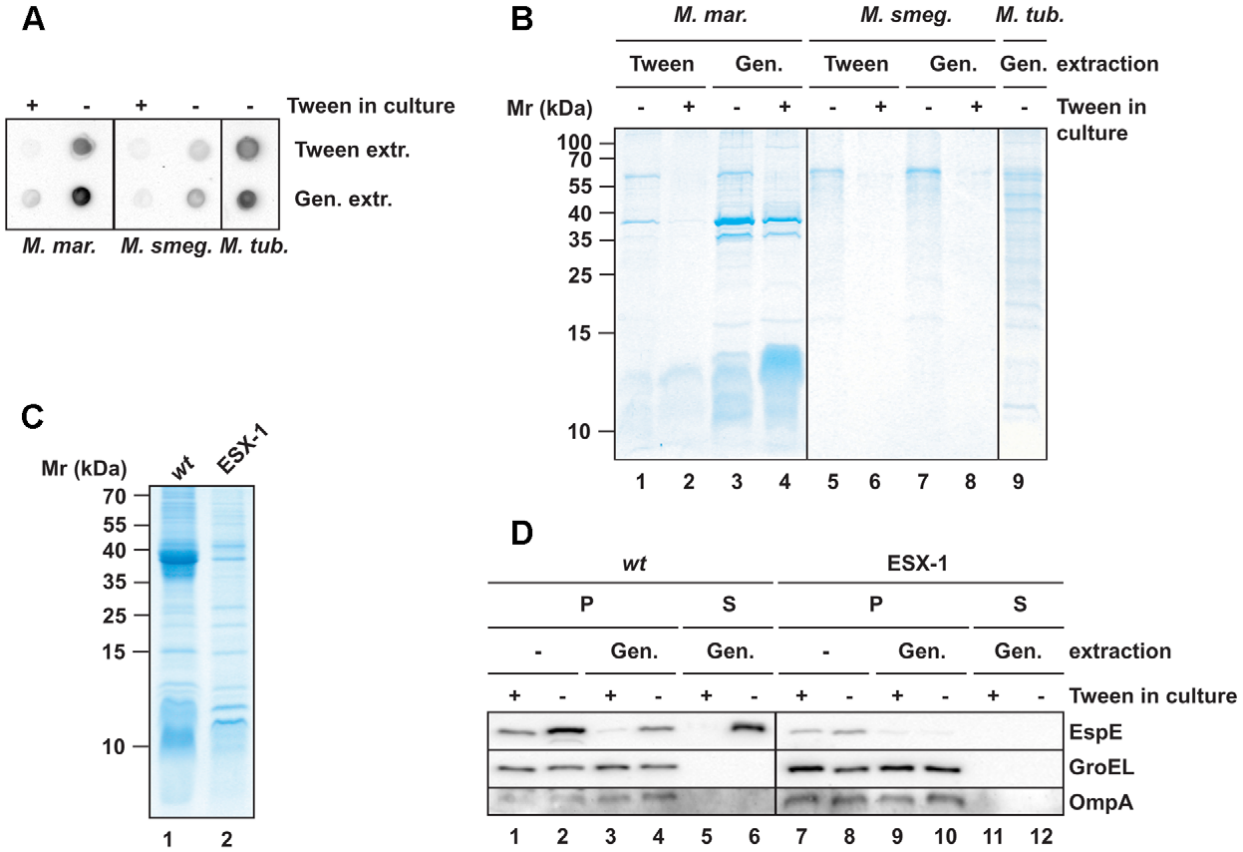
Extraction of mycobacterial capsules by mild detergent. Cell surface extracts from mycobacteria that were cultured under perturbed and unperturbed conditions (first normalized to proteins amounts of the pellet fraction) were prepared by incubation with 1% Tween-80 or 0.25% Genapol X-080. (A and B) Extracted fractions were analyzed for the presence of α-glucan by a spot blot assay (*A*) and for the protein content by SDS-PAGE and Coomassie staining (*B*). (C) Comparisons of the extracted proteins from wt *M. marinum* and an ESX-1 mutant by SDS-PAGE and Coomassie staining. (D) Immunoblot showing the Genapol-extractability of EspE. Non-extracted material (P) was separated from the extracted material (S) by centrifugation and equivalent amounts were loaded. As negative controls, the mycomembrane protein OmpA and the cytosolic protein GroEL were analyzed.

To screen for specific (ESX-secreted) proteins within the capsule for which no antibodies are available, we analyzed capsular extracts by liquid chromatography mass spectrometry (LC-MS). For this, capsular material was isolated from cells grown under unperturbed conditions by treating the cells with the mild detergents Tween-80 (1%) or Genapol-X080 (0.25%) (Figure 4). First, we analyzed the isolated fractions and the cell pellet for the presence of capsular α-glucan by a spot-blot assay. Consistent with our EM data, both *M. marinum* and *M. smegmatis* cells contained more α-glucan when they were grown in the absence of Tween-80 (Figure 4A and S5, respectively). Furthermore, spot-blot analysis showed that α-glucan was extracted by these mild detergents. These fractions were subsequently analyzed by SDS-PAGE. Extracts from bacteria grown in the absence of detergent repeatedly contained higher amounts of proteins, which indicate that their presence depends on an intact capsule (Figure 4B). In addition, more proteins were extracted by Genapol as compared to Tween.

The Genapol-extracted fractions from bacteria grown in the absence of Tween-80 were analyzed by LC-MS (Table 1). The highest number of peptides found in the *M. marinum* extract belonged to EspE, whose predicted molecular weight of 40 kDa corresponds to the size of the most abundant protein in the extract (Figure 4B). Interestingly, other ESX-1-associated proteins such as EspB, EspF (Mh3865), EspK (Mh3879c), ESAT-6 (EsxA), CFP-10 and PPE68_1 were also present in high amounts. In addition, several other PE and PPE proteins were found in the Genapol extracts. OmpA was not detected in the Genapol-extracted material, confirming that this treatment did not extract proteins from the mycomembrane. These data show that *M. marinum* has a specific set of proteins within its capsule.

**Table 1:** The 25 major cell surface extracted proteins of *M. marinum*.

*Gene*	*Spectral counts*		*Description*
	*wt*	*ESX-1*	
MM5439	238	6	EspE
MM1129	99	79	PPE family protein
MM1402	89	166	PPE family protein
MM2459	83	57	PE_PGRS30
MM5440	71	17	EspF
MM3962	57	24	fatty acid synthase Fas
MM5449/0187*	56	29	EsxB/EsxB_1
MM1014	56	32	iron-regulated elongation factor Ef-tu Tuf
MM0186	48	1	PPE68_1
MM0759	48	53	chaperonin GroEL2
MM5047	42	80	PPE64
MM4089	37	21	ATP synthase alpha chain AtpA
MM5455	35	1	EspK
MM5364	34	16	polyketide synthase Pks13
MM1126	33	44	chaperonin GroEL1
MM1179	33	32	oxidoreductase
MM2586	31	29	conserved secreted protein
MM2914	30	7	catalase-peroxidase-peroxynitritase T KatG
MM0476	29	20	conserved membrane protein
MM0996	28	14	DNA-directed RNA polymerase beta chain RpoC
MM1767	28	7	multifunctional mycocerosic acid synthase membrane-associated Mas
MM2821	28	4	isocitrate lyase AceAb
MM1497	27	56	PPE family protein
MM5457	27	2	EspB
MM5450/0188	26	3	EsxA/EsxA_1

The situation was different for *M. smegmatis* and *M. tuberculosis*. The capsular extracts of these two species did not show any major protein bands (Figure 4B). This observation was confirmed by LC-MS analysis, which showed that the major proteins were cytosolic proteins such as GroEL2, DnaK and translation elongation factor Tu (Table S2 and S3). The presence of some of these proteins in the capsule was also recently described by Hickey *et al*. [29]. In addition to these predominantly cytosolic proteins only very small amounts of putative capsular proteins, such as the ESX-1-associated proteins, were identified by LC-MS (data not shown). This could indicate that the capsular proteins of *M. smegmatis* and *M. tuberculosis* might be more tightly associated with the cell envelope than in *M. marinum* and are therefore missed during analysis.

### The major capsular proteins of *M. marinum* are secreted via ESX-1

The capsule of *M. marinum* contains a high amount of ESX-1 associated protein. To investigate whether these proteins depend on ESX-1 for their localization, Genapol extracted proteins of the *M. marinum* Fas 3.1 *eccCb1* mutant were analyzed (Figure 4C). The protein pattern of the ESX-1 mutant differed considerably from the wild-type protein pattern. Importantly, the 40 kDa product corresponding to EspE was not detectable in the capsular extract of the ESX-1 mutant (Figure 4C and D), suggesting that the capsular localization of EspE depends on an active ESX-1 secretion system in *M. marinum* as previously reported [26]. This result was also confirmed by LC-MS analysis (Table 1) and shows that EspF, EspK and the PPE proteins associated with the ESX-1 pathway are dependent on an active ESX-1 system for capsular localization and are therefore new putative ESX-1 substrates. The presence of other PE and PPE proteins in the capsule was not affected by the ESX-1 mutation, which is in agreement with the recent observation that many of these proteins, including the capsule PPE protein MMAR_1402, depend on another secretion system, ESX-5 for their export [30].

### Capsular layer promotes binding to macrophages and inhibits pro-inflammatory cytokine response

**Figure 5:**
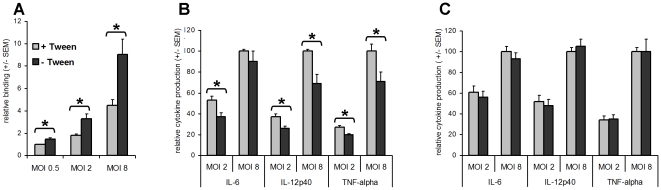
Capsular layer promotes binding to macrophages and inhibits the pro-inflammatory cytokine response. The results represent the mean relative binding ± standard error of the mean (SEM) of the pooled data from three independent donors. (A) The percentage of macrophages associated with Tween. *M. bovis* BCG expressing dsRed bacteria was determined using flow cytometry. Binding of Tween-treated *M. bovis* BCG at MOI 0.5 (1.13% +/− 0.24 (SD)) is set at 1. (B and C) Macrophages were stimulated with/without Tween treatment *M. bovis* BCG (B) or *M. smegmatis* (C) at MOIs 2 and 8. Cells stimulated with PBS served as a control. After 24 h, the supernatants were harvested and the amount of IL-12p40, IL-6, and TNF-α was determined using ELISA. The results represent the mean relative cytokine production (± SEM) of the pooled data from at least three independent donors. Cytokine production following stimulation with Tween-treated bacteria at MOI 8 is set at 100 [for *M. bovis* BCG, IL-6: 12087±3056, IL-12p40: 2928±618, TNFα: 38490±6446; for *M. smegmatis*, IL-6: 61629±17411, IL-12p40: 22693±2100, TNFα: 122663±17350 (all in pg mL^−1^ ± SEM)]. An asterisk indicates significant differences (*p*<0.05).

As demonstrated above, growing mycobacteria in the presence of detergent with agitation, promotes capsular shedding and thus may influence the biological characteristics of the bacteria. To investigate this issue and to potentially gain insight into the biological relevance of the capsule, *M. bovis* BCG was grown under unperturbed conditions after which the bacteria were treated with 1% Tween-80 to remove the capsule. First, we investigated the consequence of capsule removal on the ability of the bacteria to bind to human monocyte-derived macrophages. As shown in Figure 5A, removal of the capsule significantly reduced bacterial binding at all tested multiplicities of infection (MOIs), suggesting that the presence of the capsule promoted association to the macrophages. Next, we investigated whether the presence of a capsule may also differentially modulate the macrophage pro-inflammatory cytokine responses. As shown in Figure 5B, non-detergent-treated *M. bovis* BCG induced significantly lower amounts of IL-12p40, IL-6, and TNFα (∼30%) as compared to the detergent-treated bacteria at both MOIs tested. The only exception was IL-6, for which the difference at MOI 8 was non-significant. Reduced cytokine induction by untreated (encapsulated) *M. bovis* BCG was observed in both resting macrophages (Figure 5B) and in macrophages primed with LPS (data not shown). Interestingly, treatment with detergent did not differentially affect the macrophage response for non-pathogenic *M. smegmatis* (Figure 5C). Overall, these findings demonstrate that at least for *M. bovis* BCG the capsule is involved in facilitating macrophage infection and, simultaneously, plays a role in dampening the pro-inflammatory macrophage response. Furthermore, these data imply that growing mycobacteria in the presence of detergent, as is done under many standard laboratory circumstances, may have important consequences for the biological characteristics of these bacteria.

### Summary and biological implications

In this study we used a plunge freezing method [18] that avoids artifacts introduced by chemical fixation and thus gives a close to native estimation of the thickness for these *in vitro* grown bacteria. We demonstrated the presence of the capsular layer in the cell envelope of both pathogenic and nonpathogenic mycobacterial species and determined its thickness to be around 30 nm (Table S1), taking into account the instability of the capsule *in vitro*; both groups have been previously shown to differ in the amount of extracellular material found in static and non-detergent-treated medium, which is interpreted as representing part of the capsule shed from growing cells [10],[11]. One possibility of circumventing this instability is to measure this layer inside phagocytic cells in the vitreous state. It is however currently very difficult to freeze large mammalian cells under physiological conditions in a vitreous state [31].

The detection of capsular components α-glucan, arabinomannan, and oligo- and poly-mannosylated compounds on the surface of whole cells by immuno gold-EM is in agreement with chemical data about the nature of the capsule [4],[10],[11]. The presence of PIMs exposed at the surface of mycobacteria was already demonstrated [32],[33],[34], however here we showed that PIM, α-glucan, and ESX secreted proteins are accessible to antibodies on intact bacteria. Mycobacterial α-glucan is implicated in the regulation of phagocytosis [35],[36], modulation of immune response [1],[21], and persistence of infection [37]. In this study we have shown that binding to macrophages and a dampening of the pro-inflammatory cytokine response is observed for encapsulated *M. bovis* BCG, which suggests that components of the capsule, such as glycans, play a role in immune response.

**Figure 6:**
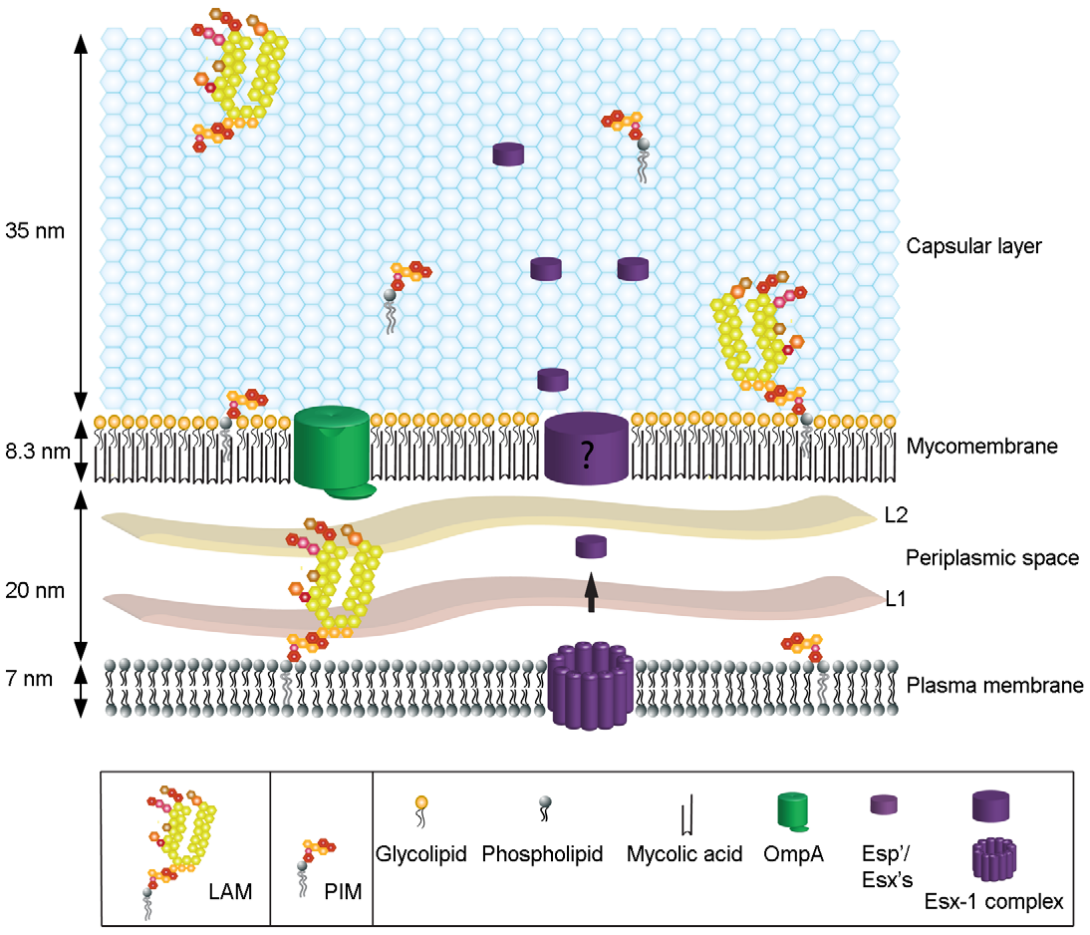
The spatial organization of the mycobacterial cell envelope exhibiting the capsule. This scheme represents the relative size and organization of the different layers of the envelope including the plasma membrane, mycomembrane, periplasmic space and capsular layer. The positions of some of the constituents analyzed are depicted.

The identification of many ESX-5 and ESX-1 secreted proteins (ESAT-6 and CFP-10, EspB, EspE, EspF, EspK and an ESX-1 associated PPE) in the capsule, a number of which have not been identified previously as being secreted via ESX-1 (EspF, EspK and PPE68_1) was surprising. These proteins were only detectable in the detergent-labile capsule fraction of *M. marinum*. Some ESX-1-secreted proteins have an essential role in the interaction with the host [38],[39],[40], and more specifically in bacterial translocation from the phagosome into the cytosol [41]. The fact that EspE can only be partly removed by Tween-80, suggests a distinction between proteins that are freely associated with the capsule and those more tightly associated with the rest of the cell envelope. In this context, these ESX-1 associated proteins may form a surface-exposed macromolecular structure through (and/or within) the capsule, specialized for the interaction with the host (Figure 6). Together, we showed that the mycobacterial capsule contains various components that manipulate the host, making this layer an attractive target for vaccine and drug development.

## Materials and Methods

### Bacterial strains and growth conditions

*M. tuberculosis* 6020 strain [42], *M. bovis* BCG strain Copenhagen, *M. smegmatis* mc2155, *M. smegmatis*-OmpATb [20], *M. marinum* E11 strain and the E11 fas 3.1 *eccCb1* mutant (van der Sar et al, in preparation) were grown at 37°C (or 30°C for *M. marinum*) in Middlebrook 7H9 media (Difco) supplemented with 10% oleic acid-albumin-dextrose catalase (OADC) (BBL) with or without 0.05% Tween 80 where applicable. For the growth of *M. smegmatis*-OmpATb and *M. bovis* BCG expressing dsRed, 25 µg and 50 µg mL^−1^ kanamycin and 50 µg mL^−1^ hygromycin were added, respectively. *S*. *flexneri ipaC* strain SF621 [43] was grown in tryptic casein soy broth (Sigma) at 37°C. *S*. *epidermidis* was grown in Luria-Bertani broth (Difco) at 37°C.

### Cryo-EM

Vitrification, vitreous sectioning and tomography is as described in *Supplementary information (SI) Text* and [44]. For plunge freezing, bacteria were taken directly from culture medium for processing without further centrifugation. A 4 µl droplet of various mycobacteria sample were applied to a glow-discharge quantifoil copper grids (Quantifoil Micro Tools, Jena, Germany) mounted in an environmentally controlled chamber at 100% humidity, blotted and frozen in vitreous ice by plunging into liquid ethane using the vitrobot (FEI). Grids were transferred to a Gatan model 626 cryoholder (Gatan, Pleasanton, CA) under liquid nitrogen and inserted into a Tecnai 12 (FEI, Eindhoven, Netherlands) operating at 120 kV. The vitreous state of the preparation was confirmed by electron diffraction. Low-dose images, with exposures between 10 and 20 electrons per Å^2^ and under-focus values of 2 to 4 µm were recorded with a 4096×4096 pixel CCD camera (Gatan) at ×18,000 −×23,000 magnification.

### Immuno gold-EM

Bacteria cells were fixed by addition of an equal volume of 0.4M PHEM buffer containing 4% paraformaldehyde and 0.4% glutaraldehyde to the culture and incubating for 2 hours at room temperature [41].

For whole mount cells, samples were taken directly from the fixed culture without pelleting and incubated for 5 minutes on carbon coated formvar grids. The rabbit antibody used is OmpATb [19]. The following murine IgM monoclonal antibodies (Mabs) were used: anti-Ara_6_ (F30-5) recognizes Ara_6_-motive in the arabinan domain of (L)AMs [45],[46]; anti-ManLAM (55.92.1A1) binds the mannosyl caps on mannosyl-capped (L)AMs [46]; anti-PIM_6_ (F183-24) recognizes terminal α(1,2)-linked mannosyl residues as present in PIM_6_ and the mannosyl caps on ManLAM [25],[45]. In *M. smegmatis* [which lacks the mannosyl-caps on (L)AM] F183-24 specifically recognizes PIM_6_ with no reactivity to other components [25]. The Mab against capsular α-glucans was generated by O. Baba [47]. Immuno-labeling is essentially as described [see *Text S1* and [41]]. Labeled whole mount cells were observed without further staining with a Tecnai 12 or CM 10 electron microscope (FEI, Eindhoven, Netherlands).

Cryo-sectioning and immuno gold labeling of fixed cells is essentially as described in *Text S1* and [41].

### Capsule extraction

Mycobacteria were grown under conditions mentioned above to an OD600 of ∼1, diluted 100 times in 7H9 without supplement or Tween-80, but with 0.2% dextrose and grown to exponential phase. Bacteria were harvested by centrifugation and washed 3 times in PBS. Bacteria were resuspended in PBS and incubated with 1% Tween-80 or 0.25% Genapol X-080 for 30 min. at room temperature before centrifugation to separate extracted fractions from bacteria cells. To determine the presence of α-glucan the extracted fractions were spotted on a nitrocellulose membrane, the membrane was dried for 1 hour at 80°C, blocked by 5% skimmed milk in PBS-T overnight and immuno-labeled using an anti-α-glucan monoclonal antibody. For analysis of the protein content, the extracted fraction was concentrated by TCA precipitation and analyzed via SDS-PAGE and Coomassie staining or Western blotting using anti-EspE [26].

### Sample preparation for mass spectrometric analysis

Protein lanes from Coomassie stained SDS-PAGE gels were excised and prepared for LC-MS analysis as described in *Text S1*.

### Preparation of mycobacteria for macrophage-binding and stimulation assays

*M. bovis* BCG, *M. bovis* BCG expressing dsRed, or *M. smegmatis* were grown in Middlebrook 7H9 broth (Difco) supplemented with 10% ADC enrichment in the absence of Tween-80. Exponentially growing bacteria were collected and washed once with 50 ml PBS. The bacteria were resuspended in 20 ml PBS, split into two 10 ml fractions, and incubated with/without 1% Tween-80 for 30 min with rotation at room temperature. The treated and untreated bacteria were washed twice with 25 mL Tris-buffered saline supplemented with 1 mM MgCl_2_ and 2 mM CaCl_2_ (TSM) containing 0.5% human serum albumin (HSA), passed through a 5 µm filter (Millipore) for declumping (bacterial suspensions were first centrifuged for 10 min at 200×*g* to prevent filter-clogging), and diluted with TSM/0.5% HSA to the appropriate optical density. Colony forming units were determined by plating the suspensions on 7H10 agar supplemented with 10% Middlebrook OADC.

### Macrophage binding assay

*M. bovis* BCG expressing dsRed was added to 5×10^5^ macrophages [generated as described in *Text S1* and ref.[21]] in 100 µL TSM/0.5% HSA at a MOI of 8, 2, or 0.5. After 45 min at 4°C, cells were washed twice with TSM/0.5% HSA and the percentage of fluorescent cells was determined using a FACScan Analytic Flow Cytometer (Becton Dickinson). Data was analyzed using manufacturer's software (CellQuest version 3.1f).

### Macrophage stimulation assay

Macrophages were released by trypsiniation and resuspended in culture medium (containing 50 U mL^−1^ GM-CSF) at a concentration of 1.25×10^6^ cells mL^−1^. Eighty µl cell suspension (1×10^5^ cells) was transferred to a sterile 96-well U-bottom plate (Greiner) and left for 16 h (37°C, 5% CO_2_) to allow cells to adhere. Macrophages were stimulated with *M. bovis* BCG or *M. smegmatis* (treated with or without 1% Tween-80) in the absence or presence of 20 ng mL^−1^ lipopolysaccharide (LPS) (from *Salmonella enterica* serotype abortus equi (Sigma-Aldrich L5886). Unstimulated cells served as controls in all experiments. Culture supernatants were harvested after 24 h of incubation (37°C, 5% CO_2_) by centrifugation and stored at −80°C for cytokine measurements using an enzyme-linked immunosorbent assay (ELISA) according to the manufacturer's instructions (Invitrogen). Data were statistically analyzed using a Student's t test (two-tailed, two-sample equal variance). Differences were considered to be significant when *p*<0.05.

## Supporting Information

Text S1 This supporting information file contains extra materials and methods
(0.05 MB DOC)

Figure S1 Mycobacteria have Gram-negative cell envelope morphology. Cultured cells were resuspended in 20% dextran for high pressure freezing before sectioning at a nominal thickness of 30 nm. Cryo-electron micrographs of vitreous cryosections of *S. flexneri* (A), *S. epidermidis* (B) *M. smegmatis* (C) show their various membrane profiles. (D-F) 10 nm thick slice from a tomographic reconstruction corresponding to A-C slightly denoised with a median filter to show the various layers of the cell wall. Tomographic slice of *S. flexenri* bacteria (D) illustrates the bilayer nature of both the plasma membrane (magenta arrow head) and the outer membrane (dark blue arrow head) typical for a bona fide Gram-negative morphology and shows a strong morphological similarity to the cell envelope profile observed for *M. smegmatis* (F); periplasmic layers 1 and 2 (L1 and L2) are denoted by white arrow heads. The slice in (E) shows a morphology typical for Gram-positive bacteria and depicts a plasma membrane (magenta arrow head) tightly bound by a thick and amorphous peptidoglycan layer (PG black arrow) with no apparent outer layer as is in D and F. Schematic drawing of the cross section of the corresponding cell envelope morphologies of D-F is depicted in D'-F'. Plasma membrane (PM; magenta arrow head) outer membrane (OM; dark blue arrow head) peptidoglycan layer (PG black arrow). Scale bars: 50 nm.
(2.41 MB TIF)

Figure S2 Vitreous sections of other mycobacteria strain. Cryo-EM images of 30 nm frozen hydrated vitreous sections of *M. tuberculosis, M. marinum* and *M. bovis* BCG vaccine strain. Scale bars: 50 nm.
(0.40 MB TIF)

Figure S3 Plot profiles of vitreous sections. Plot profiles of the cryo-EM images of 30 nm vitreous section depicted in Figure 1 shows the multilayered spatial organization of the cell envelope of *S.flexneri* (A), *S.epidermidis* (B) and *M.smegmatis* (C). Plasma membrane (PM), granular layer (GL), outer membrane (OM), peptidoglycan layer (PG) periplasmic layers 1 and 2 (L1 and L2) and mycomembrane (MOM).
(8.53 MB TIF)

Figure S4 Localization of capsular components and the effect of detergent layer. *M. bovis* BCG (A and B) and *M. tuberculosis* (D and E) were grown under both perturbed (A and D) and unperturbed (B and E) conditions, fixed and probed with anti-arabinomannan antiserum (A-B) and anti-ManLAM antiserum (D-E). Samples were prepared by mounting whole cell on a carbon coated copper grid, labeling with antibodies and protein A-gold (10 nm) and directly imaged without any staining. Histogram (C and F) displays the variations in arabinomannan (C) and ManLAM (F) labeling of bacteria due to change in the culturing conditions. The ordinate (defined as percentage of bacteria with ≥10 gold particles) represents the average percentage ± standard error of labeled cells from 3 independent experiments. Scale bars: 250 nm.
(1.32 MB TIF)

Figure S5 Dot blot assay demonstrates presence of α-glucan. The amount of α-glucan present on bacterial cells were analysed by a spot blot assay for both *M. marinum* and *M. smegmatis* and shows that these cells contain more α-glucan when they were grown in the absence of Tween-80 as compared to cultures that were grown with Tween-80, consistent with a more intact capsule layer in the absence of detergent.
(1.14 MB TIF)

Table S1 Measurement of bacteria cell envelope compartments
(0.04 MB DOC)

Table S2 The 25 major cell surface extracted proteins of *M*. *tuberculosis*
(0.04 MB DOC)

Table S3 The 25 major cell surface extracted proteins of *M*. *smegmatis*
(0.04 MB DOC)
