# Supplementary material for: Direct Visualization by Cryo-EM of the Mycobacterial Capsular Layer: A Labile Structure Containing ESX-1-Secreted Proteins
Source: PLoS Pathog. 2010 Mar 5;6(3):e1000794. doi: 10.1371/journal.ppat.1000794 (PMC2832766; doi:10.1371/journal.ppat.1000794)
Supplement: Text S1 — This supporting information file contains extra materials and methods (0.05 MB DOC) [file ppat.1000794.s001.doc]

***SI Materials and Methods***

**Vitrification and cryosectioning**

For cryo-electron microscopy of vitreous sections, cells in growth medium were mixed in an equal volume of 40 % dextran (40 kDa, Sigma- Aldrich) as cryoprotectant. Samples were drawn into copper tubes and vitrified with an EMPACT1 high pressure freezer (LEICA). Copper tubes were mounted in an FC6 cryo-ultra-microtome with a cryosphere (LEICA) at a temperature of -150°C. Cryosections were produced at a nominal thickness of 30 nm with a 45° diamond knife (Diatome) essentially as previously described [1] and placed on C-flat grids (Protochips Inc., Raleigh, NC, USA) with 10 nm fiducial gold deposited on it. Grids of vitreous sections were transferred to a Gatan model 626 cryoholder (Gatan, Pleasanton, CA) under liquid nitrogen and inserted into a Tecnai 12 (FEI, Eindhoven, Netherlands) operating at 120 kV. The vitreous state of the preparation was confirmed by electron diffraction. Low-dose images, with exposures typically between 10 and 20 electrons per Å^2^ and under-focus values of 2 to 4 μm were recorded with a 4096 x 4096 pixel CCD camera (Gatan) at x 18,000 – x 23,000 magnification. For cryo-electron tomography, single axis tilt series were acquired at an angular range of ± 60° with 2° increments using Xplore 3D automated data acquisition software (FEI, Eindhoven, Netherlands). The total dose was kept at approximately 60 e/ Å^2^. The nominal under focus value was -3.5 μm and the pixel size at the specimen level was 0.46 nm.

**Image processing**

Average density profiles of 2D images were calculated along rectangular selections with the ImageJ software (NIH, Bethesda, MD). Alignment and reconstruction of the tilt series were performed with IMOD software [2]. Tilt series were aligned by using 10 nm fiducial gold markers. Three dimensional reconstructions were calculated by weighted back projection. Reconstructed tomograms were denoised by non-linear anisotropic diffusion [3].

**Immuno-EM of whole mount cell and sections**

For Immuno-labeling of whole mount cells, grids were floated at room temperature on drops of 3x phosphate-buffered saline (PBS) pH 7.3, blocked with PBS-BSA 1% for 3-5 min; incubated with antibodies (at a concentration of 1/5 to 1/100 diluted in PBS/1% BSA) for 1 h; washed 3 times in PBS/0.15 M glycine and 5 times in PBS/0.1% BSA. Antibodies were then labeled with rabbit anti-mouse bridging serum (DAKO) (if monoclonal) and probed with protein-A conjugated to 10 nm gold (EM laboratory, Utrecht University).

For sectioning, fixed cells were embedded in gelatin, cooled on ice and cut into cubes. Cubes of cells were cryo-protected in 2.3 M sucrose trimmed using a diamond Cryotrim 90 ° knife at –100 °C (Diatome, Switzerland) and ultrathin sections of 50-70 nm were cut at –120 °C using a Cryoimmuno knife (Diatome, Switzerland). Sections on carbon coated formvar grids were labeled essentially as above and stained with 2% methylcellulose with 2% uranyl acetate to enhance contrast.

**Generation of human monocyte-derived macrophages**

Monocyte-derived macrophages were generated from human peripheral blood mononuclear cells (PBMCs) PBMCs were isolated from heparinised blood from healthy volunteers (Sanquin blood bank Amsterdam, Amsterdam, The Netherlands) using density-gradient centrifugation over a Ficoll gradient (Amersham Biosciences). PBMC fractions were washed six times with 50 ml cold PBS containing 0.5% sodium citrate (w/v). Next, monocytes were isolated from PBMCs by a CD14 selection step using the MACS Midi system (Miltenyi Biotec GmbH). Monocytes were differentiated into macrophages in RPMI-1640 medium supplemented with 10% fetal calf serum (FCS), 100 units mL^-1^ penicillin and 100 µg mL^-1^ streptomycin (all from Invitrogen) in the presence of 50 U mL^-1^ granulocyte/macrophage colony-stimulating factor (GM-CSF) (PeproTech Inc). Fresh GM-CSF was added after three days of culture. At day six or seven, the macrophages were harvested by scrapping or trypsinisation and used for further experiments.

**Mass spectrometric analysis**

Protein lanes from Coomassie stained SDS-PAGE gels were excised and prepared for LC-MS analysis as previously described [4,5]. nanoLC-MS/MS was performed on an LTQ-FT mass spectrometer (ThermoFisher, Bremen, Germany) coupled on-line to an U3000 nanoLC (Dionex/LC PAckings, Amsterdam, The Netherlands) and a nanomate ESI chip interface (Advion, Ithaca, NJ), for LC-MS details see [4]. MS/MS spectra were searched against the *M. marinum*, *M. tuberculosis* (H37Rv) and *M. smegmatis* FASTA databases using Sequest (version 27 rev12) with methionine oxidation and cysteine carboxamidomethylation as variable modifications and allowing a maximum peptide mass deviation of 10 ppm and a fragment mass deviation of 1 amu. Peptide identifications were validated using Scaffold (version 2.01.01). Proteins were identified with minimally 2 unique peptides, PeptideProphet [6] scores of >95% and a ProteinProphet [7] score of >99%. Label-free relative quantitation was performed by spectral counting. For each identified protein the number of associated MS/MS spectra was counted and used as quantitative value (Table 1), spectral counts have been shown to be proportional with relative protein abundance in a complex mixture [8].

1. Pierson J, Fernandez JJ, Bos E, Amini S, Gnaegi H, et al. (2009) Improving the Technique of Vitreous Cryo-Sectioning for Cryo-Electron Tomography: Electrostatic charging for Section Attachment and an Anti-contamination Box. J Struct Biol: In press.

2. Kremer JR, Mastronarde DN, McIntosh JR (1996) Computer visualization of three-dimensional image data using IMOD. J Struct Biol 116: 71-76.

3. Frangakis AS, Hegerl R (2001) Noise reduction in electron tomographic reconstructions using nonlinear anisotropic diffusion. J Struct Biol 135: 239-250.

4. Piersma SR, Broxterman HJ, Kapci M, de Haas RR, Hoekman K, et al. (2009) Proteomics of the TRAP-induced platelet releasate. J Proteomics 72: 91-109.

5. Shevchenko A, Wilm M, Vorm O, Mann M (1996) Mass spectrometric sequencing of proteins silver-stained polyacrylamide gels. Anal Chem 68: 850-858.

6. Keller A, Nesvizhskii AI, Kolker E, Aebersold R (2002) Empirical statistical model to estimate the accuracy of peptide identifications made by MS/MS and database search. Anal Chem 74: 5383-5392.

7. Nesvizhskii AI, Keller A, Kolker E, Aebersold R (2003) A statistical model for identifying proteins by tandem mass spectrometry. Anal Chem 75: 4646-4658.

8. Liu H, Sadygov RG, Yates JR, III (2004) A model for random sampling and estimation of relative protein abundance in shotgun proteomics. Anal Chem 76: 4193-4201.
