# Supplementary figures and images for: Direct Visualization by Cryo-EM of the Mycobacterial Capsular Layer: A Labile Structure Containing ESX-1-Secreted Proteins
Source: PLoS Pathog. 2010 Mar 5;6(3):e1000794. doi: 10.1371/journal.ppat.1000794 (PMC2832766; doi:10.1371/journal.ppat.1000794)

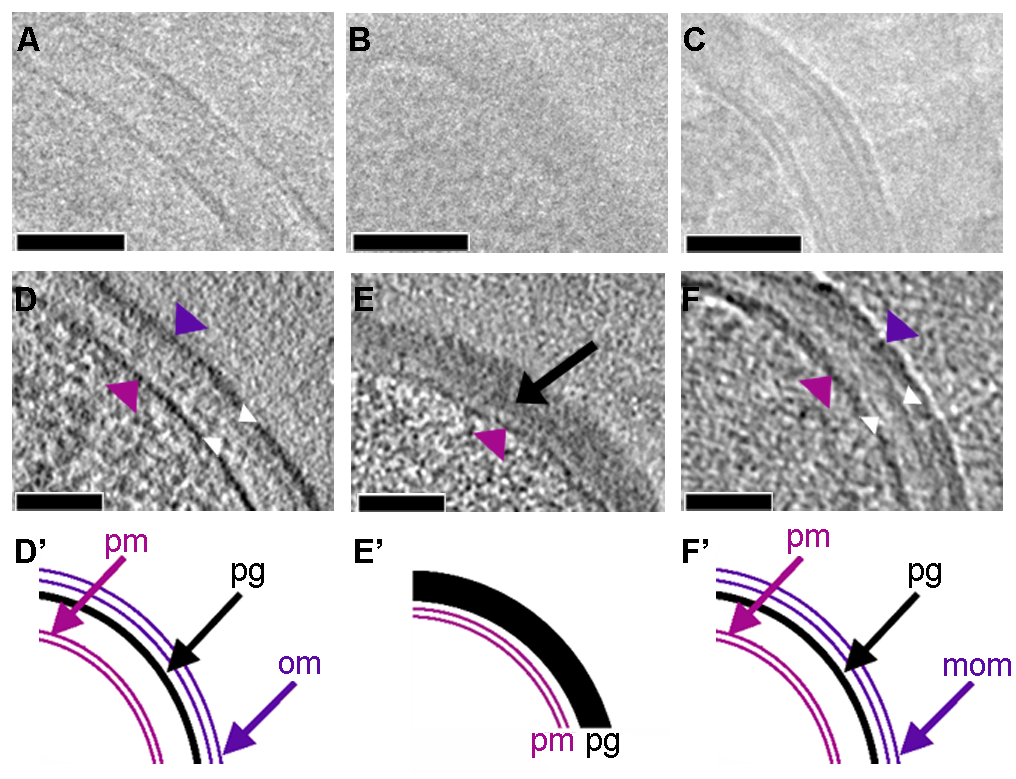

Supplement: Figure S1 — Mycobacteria have Gram-negative cell envelope morphology. Cultured cells were resuspended in 20% dextran for high pressure freezing before sectioning at a nominal thickness of 30 nm. Cryo-electron micrographs of vitreous cryosections of S. flexneri (A), S. epidermidis (B) M. smegmatis (C) show their various membrane profiles. (D-F) 10 nm thick slice from a tomographic reconstruction corresponding to A-C slightly denoised with a median filter to show the various layers of the cell wall. Tomographic slice of S. flexenri bacteria (D) illustrates the bilayer nature of both the plasma membrane (magenta arrow head) and the outer membrane (dark blue arrow head) typical for a bona fide Gram-negative morphology and shows a strong morphological similarity to the cell envelope profile observed for M. smegmatis (F); periplasmic layers 1 and 2 (L1 and L2) are denoted by white arrow heads. The slice in (E) shows a morphology typical for Gram-positive bacteria and depicts a plasma membrane (magenta arrow head) tightly bound by a thick and amorphous peptidoglycan layer (PG black arrow) with no apparent outer layer as is in D and F. Schematic drawing of the cross section of the corresponding cell envelope morphologies of D-F is depicted in D'-F'. Plasma membrane (PM; magenta arrow head) outer membrane (OM; dark blue arrow head) peptidoglycan layer (PG black arrow). Scale bars: 50 nm. (2.41 MB TIF) [file ppat.1000794.s002.tif]

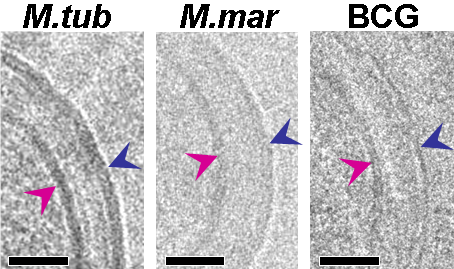

Supplement: Figure S2 — Vitreous sections of other mycobacteria strain. Cryo-EM images of 30 nm frozen hydrated vitreous sections of M. tuberculosis, M. marinum and M. bovis BCG vaccine strain. Scale bars: 50 nm. (0.40 MB TIF) [file ppat.1000794.s003.tif]

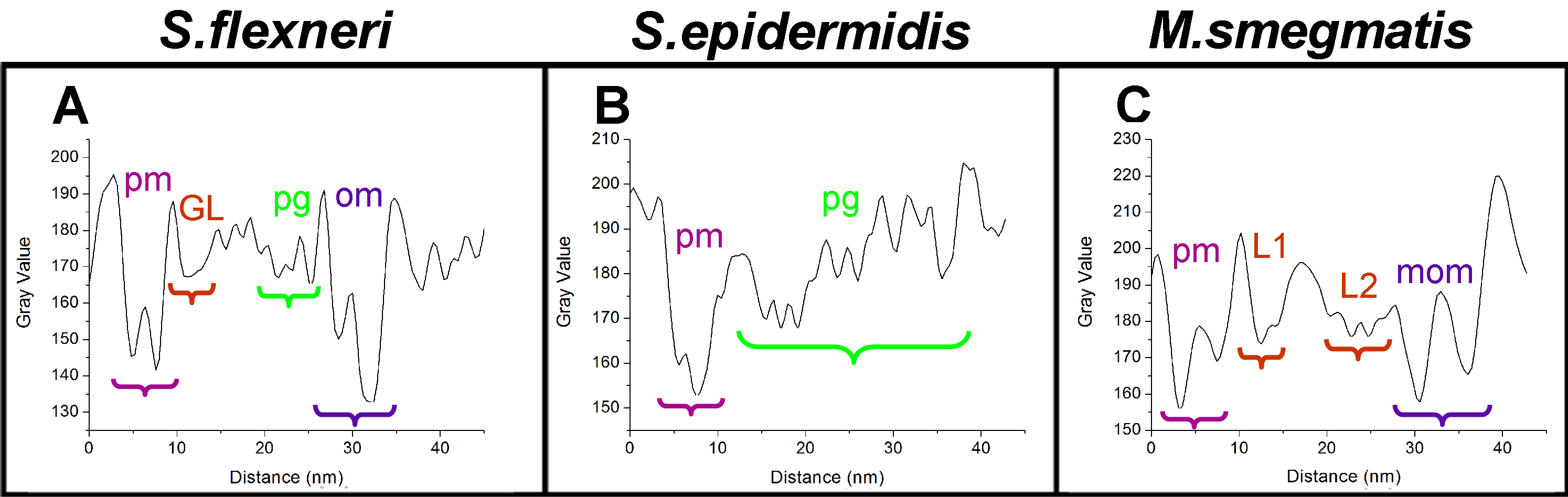

Supplement: Figure S3 — Plot profiles of vitreous sections. Plot profiles of the cryo-EM images of 30 nm vitreous section depicted in Figure 1 shows the multilayered spatial organization of the cell envelope of S.flexneri (A), S.epidermidis (B) and M.smegmatis (C). Plasma membrane (PM), granular layer (GL), outer membrane (OM), peptidoglycan layer (PG) periplasmic layers 1 and 2 (L1 and L2) and mycomembrane (MOM). (8.53 MB TIF) [file ppat.1000794.s004.tif]

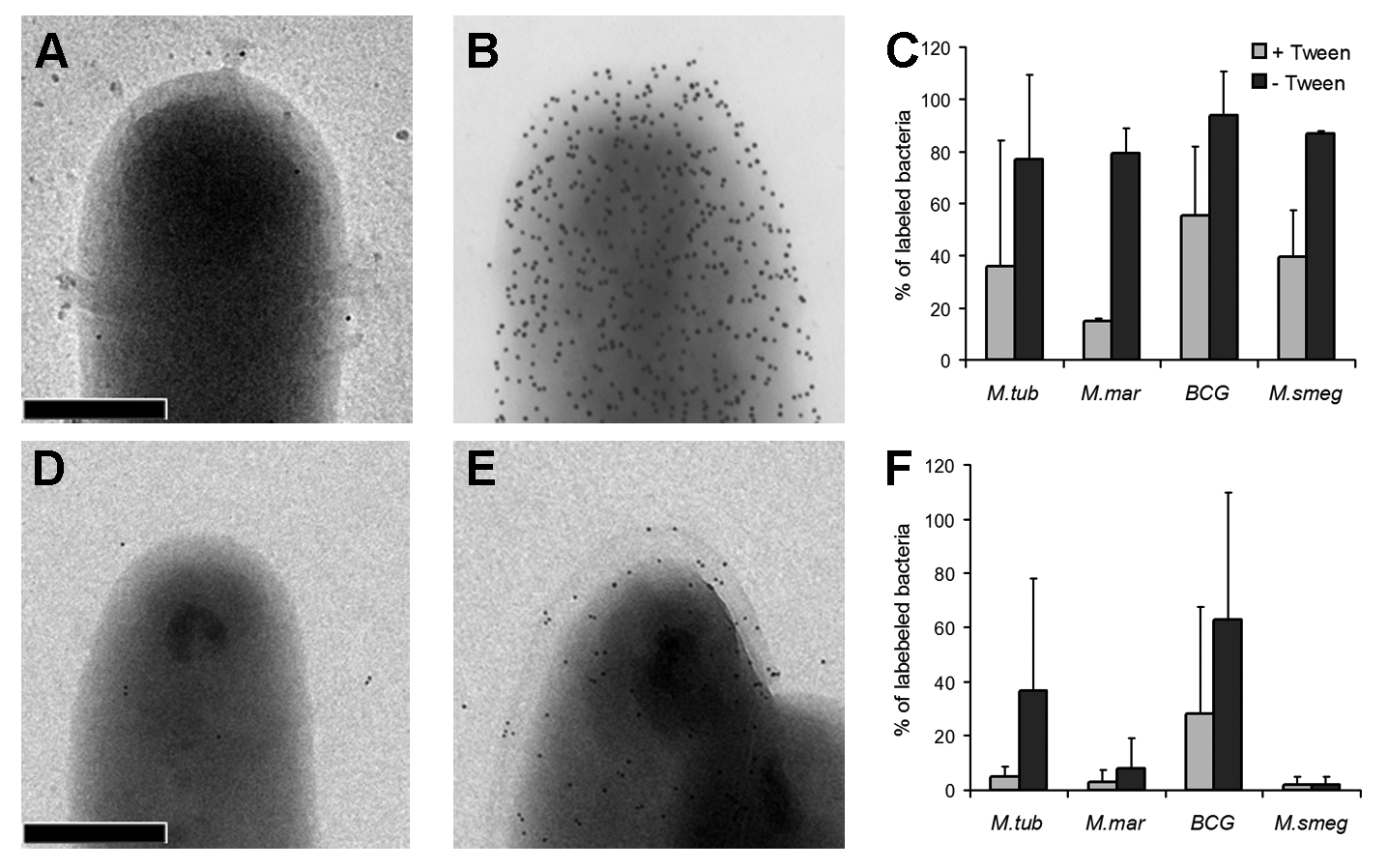

Supplement: Figure S4 — Localization of capsular components and the effect of detergent layer. M. bovis BCG (A and B) and M. tuberculosis (D and E) were grown under both perturbed (A and D) and unperturbed (B and E) conditions, fixed and probed with anti-arabinomannan antiserum (A-B) and anti-ManLAM antiserum (D-E). Samples were prepared by mounting whole cell on a carbon coated copper grid, labeling with antibodies and protein A-gold (10 nm) and directly imaged without any staining. Histogram (C and F) displays the variations in arabinomannan (C) and ManLAM (F) labeling of bacteria due to change in the culturing conditions. The ordinate (defined as percentage of bacteria with ≥10 gold particles) represents the average percentage ± standard error of labeled cells from 3 independent experiments. Scale bars: 250 nm. (1.32 MB TIF) [file ppat.1000794.s005.tif]

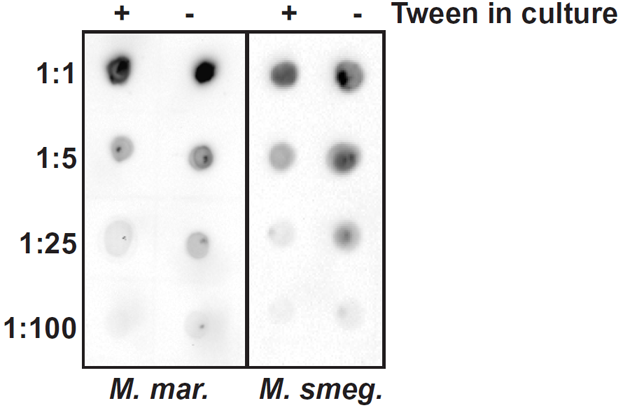

Supplement: Figure S5 — Dot blot assay demonstrates presence of α-glucan. The amount of α-glucan present on bacterial cells were analysed by a spot blot assay for both M. marinum and M. smegmatis and shows that these cells contain more α-glucan when they were grown in the absence of Tween-80 as compared to cultures that were grown with Tween-80, consistent with a more intact capsule layer in the absence of detergent. (1.14 MB TIF) [file ppat.1000794.s006.tif]
