# Supplementary material for: Direct Visualization by Cryo-EM of the Mycobacterial Capsular Layer: A Labile Structure Containing ESX-1-Secreted Proteins
Source: PLoS Pathog. 2010 Mar 5;6(3):e1000794. doi: 10.1371/journal.ppat.1000794 (PMC2832766; doi:10.1371/journal.ppat.1000794)
Supplement: Table S1 — Measurement of bacteria cell envelope compartments (0.04 MB DOC) [file ppat.1000794.s007.doc]

**Table S1.** Measurement of bacteria cell envelope compartments.

| **Thickness (nm)** ^a^ | | | | | | | |
| --- | --- | --- | --- | --- | --- | --- | --- |
| **CEMOVIS** | | | **Plunge freezing** | | | | |
| **Bacteria strain** | **PM** | **PG** | **(M)OM** | **Cell wall** ^b^ | **PM** | **(M)OM** | **Capsule** ^d^ |
| *S. epidermidis* | 5.5 ± 0.57 | NA*^c^* | NA | 31.94 ± 2.66 | NA | NA | NA |
| *S. flexneri* | 5.72 ± 0.10 | 5.6 ± 0.50 | 6.8 ± 0.30 | 22.64 ± 0.89 | NA | NA | NA |
| *M.tub* 6020 | 7.01 ± 0.25 | 5.7 ± 0.45 | 8.4 ± 0.32 | 34.56 ± 0.70 | 7.01 ± 0.1 | 8.21 ± 0.20 | 39.1 ± 3.19 |
| *M. marinum* | 6.95 ± 0.18 | 5.4 ± 0.48 | 8.2 ± 0.57 | 33.27 ± 0.67 | 7.06 ± 0.2 | 8.37 ± 0.15 | 38.80 ± 1.27 |
| *M. bovis* BCG | 6.98 ± 0.38 | 5.9 ± 0.48 | 8.3 ± 0.57 | 33.27 ± 0.67 | 7.05 ± 0.1 | 8.23 ± 0.21 | 36.14 ± 2.22 |
| *M. smegmatis* | 6.98 ± 0.10 | 5.6 ± 0.27 | 8.11 ± 0.6 | 33.93 ± 0.90 | 7.0 ± 0.12 | 8.22 ± 0.18 | 29.16 ± 1.71 |

^a^ Values represent the mean standard deviations for 10 measurements.

^b^ thickness corresponds to the total width of the structure calculated from surface to surface between CM and (M)OM

*^c^* NA, not applicable

^d^ values represent the mean standard deviations for more than 20 cells of which 5 different random positions were measured for each cell
