# Supplementary material for: Direct Visualization by Cryo-EM of the Mycobacterial Capsular Layer: A Labile Structure Containing ESX-1-Secreted Proteins
Source: PLoS Pathog. 2010 Mar 5;6(3):e1000794. doi: 10.1371/journal.ppat.1000794 (PMC2832766; doi:10.1371/journal.ppat.1000794)
Supplement: Table S2 — The 25 major cell surface extracted proteins of M. tuberculosis (0.04 MB DOC) [file ppat.1000794.s008.doc]

**Table S2.** The 25 major cell surface extracted proteins of *M. tuberculosis*.

| ***Gene*** | ***Spectral counts*** | ***Description*** |
| --- | --- | --- |
| Rv0440 | 165 | chaperonin GroEL2 |
| Rv1308 | 131 | ATP synthase alpha chain AtpA |
| Rv1310 | 115 | ATP synthase beta chain AtpD |
| Rv1309 | 63 | ATP synthase gamma chain AtpG |
| Rv1475c | 57 | iron-regulated aconitate hydratase |
| Rv0350 | 57 | chaperone protein DnaK |
| Rv2721c | 52 | possible conserved transmembrane alanine and glycine rich protein |
| Rv2244 | 50 | meromycolate extension acyl carrier protein AcpM |
| Rv1307 | 48 | ATP synthase delta chain AtpH |
| Rv1006 | 48 | hypothetical protein |
| Rv1133c | 44 | 5-methyltetrahydropteroyltriglutamate--homocysteine methyltransferase MetE |
| Rv1078 | 44 | proline-rich antigen homolog Pra |
| Rv0227c | 41 | probable conserved membrane protein |
| Rv1223 | 40 | serine protease HtrA |
| Rv0242c | 40 | 3-oxoacyl-[acyl-carrier protein] reductase FabG4 |
| Rv1837c | 39 | malate synthase G GlcB |
| Rv1488 | 39 | possible exported conserved protein |
| Rv0873 | 39 | acyl-CoA dehydrogenase FabE10 |
| Rv1836c | 38 | conserved hypothetical protein |
| Rv2744c | 35 | conserved 35 kDa alanine rich protein |
| Rv1908c | 33 | catalase-peroxidase-peroxynitritase T KatG |
| Rv0831c | 32 | conserved hypothetical protein |
| Rv2953 | 31 | conserved hypothetical protein |
| Rv2224c | 30 | probable exported protease |
| Rv2091c | 30 | probable membrane protein |
