# Supplementary material for: Direct Visualization by Cryo-EM of the Mycobacterial Capsular Layer: A Labile Structure Containing ESX-1-Secreted Proteins
Source: PLoS Pathog. 2010 Mar 5;6(3):e1000794. doi: 10.1371/journal.ppat.1000794 (PMC2832766; doi:10.1371/journal.ppat.1000794)
Supplement: Table S3 — The 25 major cell surface extracted proteins of M. smegmatis (0.04 MB DOC) [file ppat.1000794.s009.doc]

**Table S3. The 25 major cell surface extracted proteins of *M. smegmatis*.**

| ***Gene*** | ***Spectral counts*** | ***Description*** |
| --- | --- | --- |
| MSMEG_1401 | 69 | translation elongation factor Tu |
| MSMEG_6759 | 50 | glycerol kinase GlpK |
| MSMEG_0880 | 46 | chaperonin GroEL2 |
| MSMEG_3084 | 32 | glyceraldehyde-3-phosphate dehydrogenase Gap |
| MSMEG_0709 | 21 | chaperone protein DnaK |
| MSMEG_1654 | 20 | isocitrate dehydrogenase, NADP-dependent |
| MSMEG_4757 | 20 | fatty acid synthase |
| MSMEG_3811 | 19 | putative universal stress protein family |
| MSMEG_0913 | 19 | methoxy mycolic acid synthase 1 |
| MSMEG_6431 | 18 | conserved hypothetical protein |
| MSMEG_2351 | 18 | electron transfer flavoprotein, beta subunit EtfB |
| MSMEG_3143 | 18 | aconitate hydratase 1 AcnA |
| MSMEG_4290 | 18 | glutamine synthetase GlnA |
| MSMEG_1583 | 17 | chaperonin GroEL1 |
| MSMEG_1469 | 17 | ribosomal protein S8 RpsH |
| MSMEG_1365 | 17 | ribosomal protein L7/L12 RplL |
| MSMEG_5524 | 16 | succinyl-CoA synthetase, alpha subunit SucD |
| MSMEG_4891 | 15 | alkylhydroperoxide reductase |
| MSMEG_3833 | 15 | 30S ribosomal protein S1 |
| MSMEG_1019/2299* | 15 | ribonucleoside- diphosphate reductase, alpha subunit |
| MSMEG_4323 | 14 | pyruvate dehydrogenase E1 component |
| MSMEG_2374 | 14 | ketol-acid reductoisomerase IlvC |
| MSMEG_1471 | 14 | ribosomal protein L18 RplR |
| MSMEG_3050 | 13 | integration host factor |
| MSMEG_5672 | 13 | citrate synthase I GltA |
